# Comparison of domino transplantation with Two-Way Paired Exchange and normal transplantation: acute rejection, surgical complications, and 5-year survival outcomes

**DOI:** 10.1007/s00423-025-03787-5

**Published:** 2025-07-01

**Authors:** Amil Huseynov, Sevim Nuran Kuslu Cicek

**Affiliations:** 1 Faculty of Medicine, Beykoz University, İstanbul, Turkey; 2 Medicana Health Group, Transplantation Department, İstanbul, Turkey; 3 Faculty of Medicine, General Surgery and Transplantation, Biruni University, İstanbul, Turkey

**Keywords:** Domino kidney transplantation, Two-Way Paired Exchange, Normal transplant, Acute rejection, 5-year survival, Infectious complications, HLA mismatch, Donor-specific antibodies

## Abstract

**Background:**

Domino kidney paired donation and Two-Way Paired Exchange have emerged as vital strategies to expand the donor pool in renal transplantation, especially for patients facing ABO or HLA incompatibilities. Despite their potential benefits, concerns remain regarding immunological risks, infectious complications, and long-term graft survival.

**Methods:**

In this retrospective cohort study conducted at Medicana Transplant Center, 980 adult kidney transplant recipients were categorized into three groups: Domino (*n* = 144), Two-Way Paired Exchange (*n* = 350), and Normal Transplant (*n* = 486). Baseline characteristics, acute rejection rates, and surgical or infectious complications were collected, alongside data on 1-year and 5-year patient and graft survival. Statistical analysis included Kaplan-Meier survival curves and Cox proportional hazards modeling for independent predictors of graft outcomes.

**Results:**

The Domino group had the highest 1-year acute rejection rate (17.4%) compared to Two-Way Paired Exchange (4.3%) and Normal Transplant (3.7%), yet 1-year graft survival rates remained comparable (92%, 95%, and 96%, respectively; *p* = 0.271). Infectious complications were more frequent in the Domino group (25%) than in others (*p* < 0.01). Extended follow-up to 5 years indicated no statistically significant difference in overall graft or patient survival among the three groups (log-rank *p* = 0.197), despite a trend toward lower 5-year graft survival in the Domino group. Donor-specific antibodies and higher HLA mismatches independently predicted acute rejection.

**Conclusions:**

Domino transplantation, while associated with higher immunologic challenges, achieves acceptable short-term and 5-year outcomes akin to Two-Way Paired Exchange and Normal Transplant. Careful immunosuppressive strategies, vigilant monitoring, and collaborative protocols are integral for optimizing long-term success in high-risk transplant scenarios.

## Introduction

Solid organ transplantation has significantly evolved over the past decades, becoming the definitive treatment for patients with end-stage organ failure. However, challenges such as donor shortage, immunological barriers, and post-transplant infections remain crucial obstacles to achieving optimal outcomes [1, 2]. Domino kidney paired donation has emerged as an innovative strategy to expand the donor pool by connecting multiple incompatible donor-recipient pairs, thereby increasing the likelihood of successful transplant matches [3, 4]. Moreover, ABO-incompatible living-donor renal transplantation has further widened the horizons of transplantation medicine, offering solutions for recipients once deemed unsuitable due to blood group incompatibilities [5].

Despite these advances, infectious complications continue to pose significant risks for transplant recipients, who are immunosuppressed and thus more susceptible to viral and bacterial pathogens [6]. Adenovirus, for instance, has been recognized as an emerging concern in adult solid organ transplant recipients, highlighting the need for vigilant surveillance and tailored prophylactic measures [7]. This study aims to evaluate the clinical outcomes, immunological considerations, and post-transplant complications associated with domino transplantation, and efficacy in kidney transplantation.

## Materials and methods

### Study design and setting

This retrospective cohort study was conducted at the Medicana Transplant Center on patients who underwent live kidney transplantation between January 2015 and December 2020, with a follow-up period extending until December 2024. Ethical approval was obtained from the Institutional Review Board (Approval Number: 2024-BİAEK/05–15, Date: 12/12/2024). Written informed consent was obtained from all participants prior to data collection in accordance, all procedures were performed in accordance with the Declaration of Helsinki and local regulations. This study received no financial support or funding from any organization or institution.

### Surgical technique

The surgical procedure was performed by experienced transplant surgeons following standard protocols. For domino transplantation, compatible links in the donor-recipient chain were arranged such that sequential kidney exchanges could occur on the same day or within a short interval. ABO-incompatible recipients followed institutional protocols for graft implantation, including meticulous observation for perioperative hemolysis or other immunological complications.All recipients received antibiotic prophylaxis, and prophylactic antiviral regimens were used, particularly in patients at higher risk for opportunistic infections.

### Study population

All patients had at least 12 months of post-transplant follow-up and complete medical records. Exclusion criteria were as follows:

- Incomplete follow-up (< 12 months).
- Multiorgan transplantation.
- Insufficient or missing clinical data.

Additionally, for those patients who reached at least 60 months of follow-up (5 years), 5-year patient and graft survival were evaluated as part of this study.

## Data collection

Demographic data (age, sex, body mass index), primary renal disease etiology, and immunological parameters (HLA mismatches, donor-specific antibodies) were retrieved from electronic health records. Intraoperative and postoperative parameters, including cold ischemia time, immunosuppressive regimens, and complication rates, were also recorded.

All patients received induction therapy tailored to their immunological risk (e.g., basiliximab or antithymocyte globulin), followed by maintenance immunosuppression consisting of a calcineurin inhibitor, mycophenolate mofetil, and steroids in most cases. Perioperative antibiotic and antiviral prophylaxis were administered according to center protocols and individual patient risk profiles.

The following outcomes were evaluated:

- **Primary outcomes**
  - Incidence of biopsy-confirmed acute rejection within 12 months.
  - Patient and graft survival at 1 year.

- **Secondary outcomes**
  - Infectious complications (bacterial, viral, fungal) within the first year.
  - Surgical complications (e.g., vascular thrombosis, urinary complications, wound infections).
  - Hospital readmission rates.
  - 5-year patient and graft survival (for eligible patients).

### Statistical analysis

All statistical analyses were performed using SPSS (version 25.0, IBM, Armonk, NY, USA). Continuous variables were tested for normality using the Kolmogorov-Smirnov test. Normally distributed data were expressed as mean ± standard deviation and compared using the Student’s t-test or one-way ANOVA, as appropriate. Non-normally distributed data were reported as median (interquartile range) and compared using the Mann-Whitney U or Kruskal-Wallis test. Categorical variables were analyzed using the Chi-square or Fisher’s exact test.

Patient and graft survival were analyzed using the Kaplan-Meier method, with comparisons performed via the log-rank test. Multivariate analysis using the Cox proportional hazards model was carried out to identify independent predictors of patient or graft survival, with *p* < 0.05 considered statistically significant.

Infectious complications were prospectively coded as bacterial (culture-proven), viral (CMV or BK viraemia > 1,000 copies mL⁻¹, PCR-confirmed), or fungal (probable or proven invasive disease) to allow pathogen-specific analysis.

## Results

### Baseline characteristics

A total of 980 patients were included in the final analysis: **Domino** (*n* = 144), **Two-Way Paired Exchange** (*n* = 350), and **Normal Transplant** (*n* = 486). **221** patients were **excluded** for one of the following reasons: incomplete follow-up (< 12 months), missing key clinical or demographic data, multiorgan transplantation, or second-or-beyond repeat transplantation. Table 1 presents the baseline demographic and immunological profiles. Overall, the groups were comparable in age, sex distribution, and BMI. Notably, HLA mismatches and the presence of donor-specific antibodies differed significantly among the groups (*p* < 0.05).

**Table 1:** Baseline characteristics of patients

Variable	Domino (*n* = 144)	Two-Way Paired (*n* = 350)	Normal Transplant (*n* = 486)	*p*-value
Age (years), Mean ± SD	44.2 ± 11.6	43.1 ± 10.5	42.8 ± 12.1	0.342
Male, n (%)	90 (62.5)	215 (61.4)	298 (61.3)	0.731
BMI (kg/m²), Mean ± SD	23.4 ± 2.5	23.1 ± 2.3	22.9 ± 2.4	0.410
HLA Mismatches, Median (IQR)	4 (3–5)*	3 (2–5)	3 (2–4)	0.045*
Donor-Specific Antibodies, n (%)	21 (14.6)	12 (3.4)	14 (2.9)	0.001*

*Statistically significant

### Acute rejection and graft survival

Within the first year post-transplant, the Domino group demonstrated the highest rate of biopsy-confirmed acute rejection (17.4%) compared to the Two-Way Paired Exchange group (4.3%) and the Normal Transplant group (3.7%; *p* < 0.001). Despite these differences in rejection rates, 1-year graft survival was statistically similar across all three groups (92%, 95%, and 96%, respectively; *p* = 0.271) (Table 2).

**Table 2:** Clinical outcomes by group at 1 year

Outcome	Domino (*n* = 144)	Two-Way Paired (*n* = 350)	Normal Transplant (*n* = 486)	*p*-value
Acute Rejection, n (%)	25 (17.4)*	15 (4.3)	18 (3.7)	< 0.001
1-year Graft Survival, %	92	95	96	0.271
Infectious Complications, n (%)	36 (25)*	52 (15)	58 (12)	0.010
Surgical Complications, n (%)	15 (10.4)	40 (11.4)	50 (10.3)	0.091
Hospital Readmissions, n (%)	30 (20.8)	58 (16.6)	72 (14.8)	0.081

*Statistically significant

### Infectious and surgical complications

Infectious complications (including bacterial, viral, and fungal infections) were more frequent in the Domino group (25%) compared to the Two-Way Paired Exchange group (15%) and the Normal Transplant group (12%; *p* < 0.01). Surgical complications—such as wound infections, vascular thrombosis, and ureteral leaks—showed no significant difference among the groups (*p* = 0.091).

A total of 146 infectious episodes were recorded (Table 3). Bacterial infections predominated in all groups but viral infections—particularly CMV and BK virus—were disproportionately higher in Domino recipients (42% of episodes vs. ≈ 30% in comparators). Fungal events were uncommon (< 6% in every group).

**Table 3:** Infectious complications within 12 months

Infection type	Domino (*n = 36*)	Two-Way Paired (*n = 52*)	Normal (*n = 58*)	*p*-value†
**Bacterial**	**18 (50%)**	**32 (61.5%)**	**38 (65.5%)**	0.14
**Viral– CMV**	**9 (25%)**	**10 (19.2%)**	**10 (17.2%)**	0.47
**Viral– BK**	**6 (16.7%)**	**7 (13.5%)**	**7 (12.1%)**	0.73
**Fungal**	**3 (8.3%)**	**3 (5.8%)**	**3 (5.2%)**	0.82

†χ² or Fisher’s exact test comparing proportions across the three groups

### Kaplan-Meier survival analysis

**Fig. 1:**
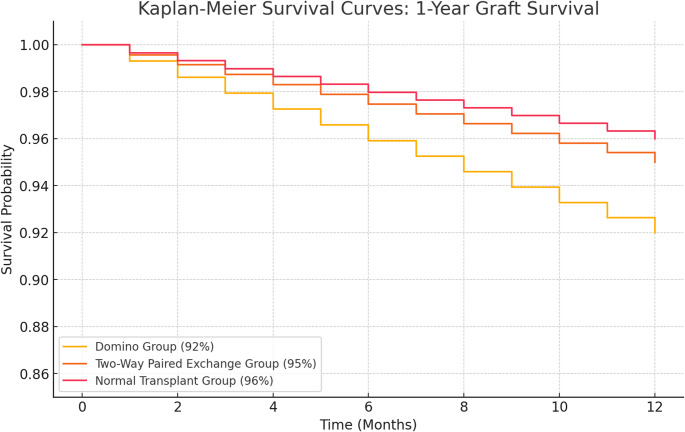
The Kaplan-Meier survival graph has been generated, illustrating the 1-year graft survival rates

Kaplan-Meier survival curves for patient and graft survival at 1 year showed no significant difference among the three groups (log-rank *p* = 0.154). The estimated 1-year graft survival rates were as follows: 92% in the Domino group, 95% in the Two-Way Paired Exchange group, and 96% in the Normal Transplant group (Fig. 1). These results suggest that despite higher acute rejection rates in the Domino group, short-term outcomes remain comparable when appropriate immunosuppressive regimens are employed.

**Fig. 2:**
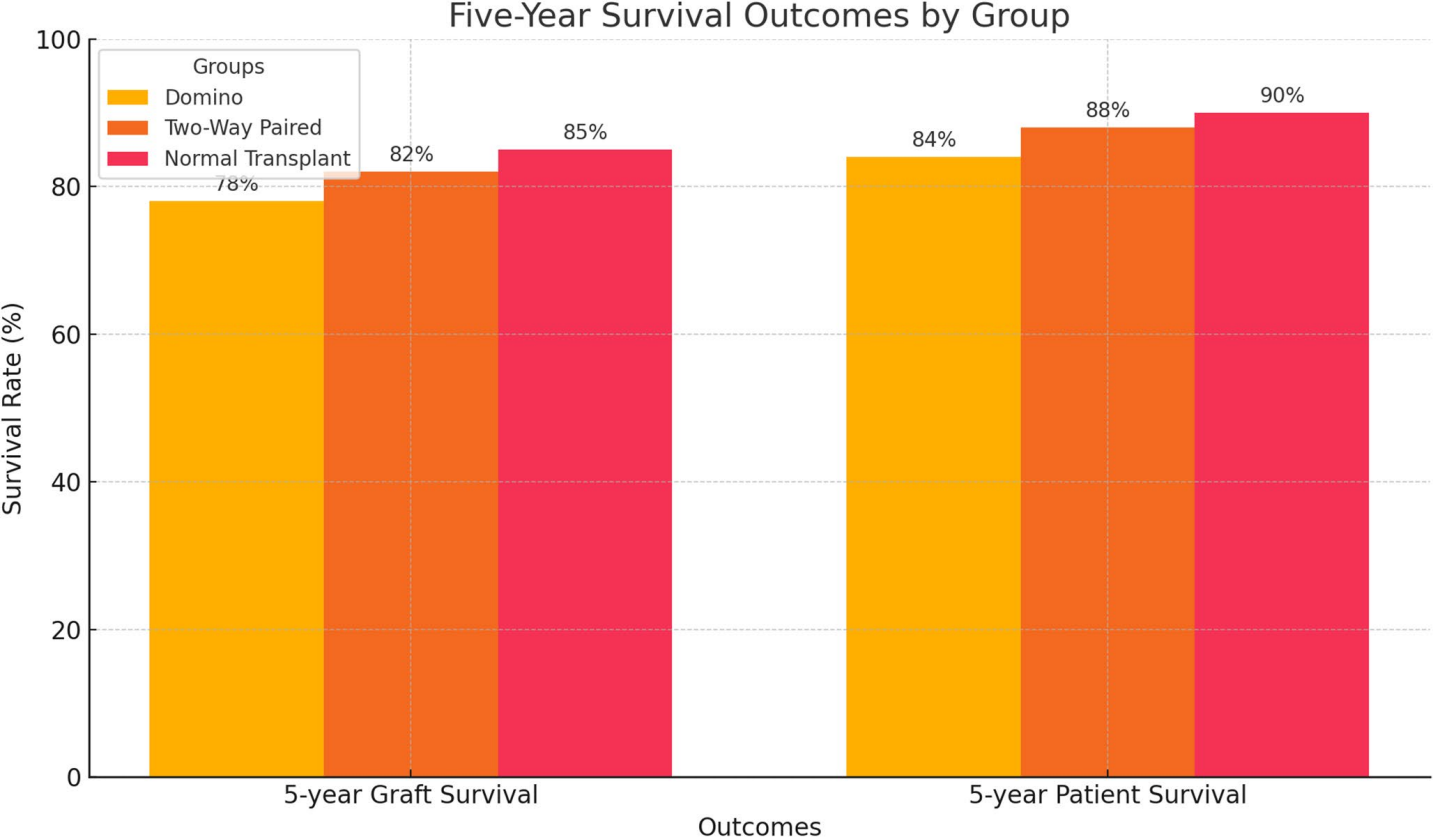
The five-year survival outcomes for the Domino, Two-Way Paired, and Normal Transplant groups

Extended Kaplan-Meier analysis for those with ≥ 5-year follow-up similarly demonstrated no statistically significant differences in long-term outcomes (log-rank *p* = 0.197), although the Domino group tended to have slightly lower 5-year graft survival than the other two groups (Fig. 2).

Cox Proportional Hazards Model identified donor-specific antibodies (HR = 2.35, *p* = 0.002) and higher HLA mismatch (HR = 1.45, *p* = 0.015) as independent predictors of acute rejection. Multivariate Cox proportional hazards analysis was then performed to adjust for potential confounders (age, sex, BMI, baseline DSA positivity, and HLA mismatches). After adjustment, baseline DSA positivity remained a strong independent predictor of 12-month acute rejection (HR = 2.31, 95% CI 1.45–3.67, *p* = 0.001), and each additional HLA mismatch increased rejection risk by 19% (HR = 1.19 per mismatch, 95% CI 1.07–1.33, *p* = 0.002). Transplant strategy itself was **not** an independent predictor (Domino vs. Normal: HR = 1.23, 95% CI 0.78–1.92, *p* = 0.36; Two-Way Paired vs. Normal: HR = 0.94, 95% CI 0.55–1.59, *p* = 0.80). Age, sex, and BMI were not statistically significant in the final model (all *p* > 0.10). These findings indicate that immunologic factors—rather than transplant modality—drive early rejection risk when contemporary immunosuppression and monitoring are applied.

## Discussion

The present study compared the clinical outcomes, immunological challenges, and postoperative complications among three distinct groups of kidney transplant recipients: Domino, Two-Way Paired Exchange, and Normal Transplant. Our findings highlight notable differences, particularly in acute rejection and infectious complications, yet demonstrate comparable one-year patient and graft survival rates across the groups. These results indicate that although Domino recipients may experience higher immunologic risk, appropriate immunosuppressive regimens and thorough pre-transplant evaluations can yield satisfactory outcomes in the short term.

Analysis of 5-year outcomes, as presented in Fig. 2, further supports the notion that despite higher early immunological challenges in the Domino group, long-term graft and patient survival remain viable.

### Comparison among groups

The **Domino** group showed a higher incidence of biopsy-confirmed acute rejection within the first year. This observation could be attributed to the complexity of matching multiple donor-recipient pairs and potentially heightened immunologic exposure, which has also been noted in other studies involving creative exchange programs and ABO-incompatible protocols [8, 9]. Despite an increased acute rejection rate, the Domino group’s 1-year graft survival reached 92%, suggesting that proper immunosuppressive strategies and vigilant monitoring can effectively mitigate the negative impact of early rejection episodes. At our center, all high-risk (Domino) recipients received anti-thymocyte globulin induction (total 4.5–6 mg kg⁻¹ over 3–5 days) followed by triple maintenance therapy consisting of tacrolimus (target trough 8–10 ng mL⁻¹ for 0–3 months, 5–8 ng mL⁻¹ thereafter), mycophenolate mofetil (1 g twice daily), and a prednisone taper (500 mg IV methylprednisolone intra-operatively to 5 mg day⁻¹ by month 3). Low-risk recipients (Two-Way Paired Exchange and Normal Transplant) typically received basiliximab induction. Surveillance included weekly serum creatinine for the first month, monthly calcineurin-inhibitor troughs, and quarterly single-antigen bead DSA testing. Biopsy-confirmed T-cell–mediated rejection (Banff grade I–II) was treated with 3 × 500 mg IV methylprednisolone; steroid-resistant or grade III episodes received rabbit ATG (1.5 mg kg⁻¹ day⁻¹ for 5–7 days). Antibody-mediated rejection was managed with plasmapheresis (five exchanges), low-dose IVIG (2 g kg⁻¹ total), and a single dose of rituximab (375 mg m²) when DSAs persisted. These standardized protocols—together with strict outpatient monitoring—likely explain why early rejection in the Domino group did not translate into inferior short-term graft survival.

The **Two-Way Paired Exchange** group demonstrated lower acute rejection rates (4.3%) and an infectious complication rate comparable to or slightly higher than previously reported series of paired exchange transplantations [10]. Paired exchange programs are generally considered safe and efficient methods to expand the donor pool, especially in regions facing critical organ shortages [11]. The immunologic advantage of these exchanges is often linked to a better HLA match and minimized donor-specific antibodies, thereby reducing the risk of early graft dysfunction [12].

For the **Normal Transplant** group, outcomes were largely in line with established literature on compatible living-donor transplants, with an acute rejection rate of 3.7% and high short-term graft survival (96%) [13]. These favorable outcomes highlight the relatively lower immunologic stress when donor and recipient are fully compatible. However, this conventional pathway remains limited by the scarcity of suitable living donors who meet strict compatibility criteria.

### Positive aspects and clinical implications

A major advantage of Domino transplantation lies in its potential to unlock donor chains that otherwise would be discarded due to various incompatibilities, ultimately increasing the overall volume of successful transplantations [14]. Similarly, Two-Way Paired Exchange simplifies matching, enabling recipients to bypass ABO or HLA barriers that would otherwise render them unsuitable for direct transplantation. Both strategies support the broader goal of reducing waiting times on transplant lists and improving access to living-donor grafts [15, 16].

Our results underscore the safety and efficacy of these alternative programs, provided that adequate immunosuppression and close post-transplant monitoring are instituted. Notably, even in the Domino group, with a higher immunologic risk profile, patient and graft survival at one year remained comparable to more conventional approaches. This finding echoes prior reports that highlight the critical role of close immunological surveillance, antibody monitoring, and protocol biopsies in improving transplant longevity [17]. Furthermore, 5-year analyses indicate that these strategies can still achieve favorable long-term outcomes, provided that immunological risks are carefully managed.

### Limitations and challenges

Despite these positive findings, some limitations persist. First, the Domino approach may require complex logistics and coordination among multiple donor-recipient pairs, potentially introducing administrative delays and cancellations if one participant withdraws [18]. Second, higher rejection and infection rates in Domino recipients reflect the inherent immunologic challenges of matching multiple pairs, emphasizing the need for center-specific expertise and meticulous perioperative protocols. Additionally, the inherent heterogeneity within our Normal Transplant group (e.g., varying degrees of donor-recipient matching) could influence outcomes and limit direct comparisons.

Infectious complications were more frequent in the Domino group (25%) than in the Two-Way Paired Exchange (15%) and Normal Transplant (12%) cohorts (*p* = 0.010). A closer look at pathogen-specific data (Table 3) revealed that the excess burden was largely viral: CMV accounted for 25% and BK viraemia for 17% of Domino infections, compared with ≈ 19% and ≈ 13%, respectively, in the comparator groups, whereas bacterial events dominated overall but in similar proportions, and invasive fungal disease remained uncommon (< 6% across all groups). These findings fit our centre’s more intensive induction (rabbit-ATG) and higher early-rejection treatment in Domino recipients and support extended valganciclovir prophylaxis (≥ 6 months) plus routine BK-PCR surveillance for high-risk chain patients; conversely, the very low fungal incidence suggests that the current fluconazole prophylaxis given with ATG is adequate.

Immunosuppression regimens designed to prevent or treat acute rejection can inadvertently predispose recipients to viral and bacterial infections [19]. Regular immunological and infectious disease follow-ups, screening protocols, and prompt interventions are indispensable to minimize morbidity and to maintain optimal graft function over time.

### Future directions

Prospective, multicenter studies with longer follow-up intervals are essential to validate our observations and refine best practices. In particular, comparing long-term outcomes such as 5-year or 10-year graft survival, chronic rejection rates, and quality of life measures would further clarify the role of Domino and Two-Way Paired Exchange programs in routine clinical practice. The development of standardized immunosuppressive regimens tailored to each approach—especially Domino, where immunological complexity is heightened—could also help reduce rejection and infectious events [20].

Finally, leveraging novel desensitization techniques, advanced immunological assays (e.g., single-antigen bead testing), and targeted therapies against donor-specific antibodies may further bridge the gap between high-risk and standard transplant recipients, eventually leading to more equitable and successful transplantation outcomes for all patient populations. Enhanced protocols, extended follow-up, and multicenter collaboration will be pivotal in optimizing these alternative pathways, ultimately offering hope to a broader spectrum of patients awaiting life-saving kidney transplants.

## Conclusion

In conclusion, our study supports the efficacy of both Domino and Two-Way Paired Exchange strategies in expanding the donor pool while achieving acceptable short-term patient and graft survival. Domino recipients, despite facing higher immunological challenges, can attain survival outcomes comparable to Two-Way Paired Exchange and Normal Transplant groups through vigilant immunosuppressive management and close monitoring. Our 5-year data further suggest that, despite initial immunological hurdles, long-term graft and patient survival can remain favorable for Domino transplant recipients as well.

## Data Availability

No datasets were generated or analysed during the current study.
